# Professionals' Views and Experiences of Using Rehabilitation Robotics With Stroke Survivors: A Mixed Methods Survey

**DOI:** 10.3389/fmedt.2021.780090

**Published:** 2021-11-11

**Authors:** Lutong Li, Sarah Tyson, Andrew Weightman

**Affiliations:** ^1^Department of Mechanical, Aerospace and Civil Engineering, School of Engineering, University of Manchester, Manchester, United Kingdom; ^2^Division of Nursing, Midwifery and Social Work, School of Health Sciences, Faculty of Biology, Medicine and Health, University of Manchester, Manchester, United Kingdom

**Keywords:** rehabilitation, robotics, home-based, clinical, stroke

## Abstract

**Objective:** To understand the reason for low implementation of clinical and home-based rehabilitation robots and their potential.

**Design:** Online questionnaire (November 2020 and February 2021).

**Subjects:** A total of 100 professionals in stroke rehabilitation area were involved (Physiotherapists *n* = 62, Occupation therapists *n* = 35).

**Interventions:** Not applicable.

**Main Measures:** Descriptive statistics and thematic content analysis were used to analyze the responses: 1. Participants' details, 2. Professionals' views and experience of using clinical rehabilitation robots, 3. Professionals' expectation and concerns of using home-based rehabilitation robots.

**Results:** Of 100 responses, 37 had experience of rehabilitation robots. Professionals reported that patients enjoyed using them and they increased accessibility, autonomy, and convenience especially when used at home. The main emergent themes were: “aims and objectives for rehabilitation robotics,” “requirements” (functional, software, and safety), “cost,” “patient factors” (contraindications, cautions, and concerns), and “staff issues” (concerns and benefits). The main benefits of rehabilitation robots were that they provided greater choice for therapy, increased the amount/intensity of treatment, and greater motivation to practice. Professionals perceived logistical issues (ease of use, transport, and storage), cost and limited adaptability to patients' needs to be significant barriers to tier use, whilst acknowledging they can reduce staff workload to a certain extent.

**Conclusion:** The main reported benefit of rehabilitation robots were they increased the amount of therapy and practice after stroke. Ease of use and adaptability are the key requirements. High cost and staffing resources were the main barriers.

## Introduction

Approximately two-thirds of stroke survivors are left with some degree of residual disability, particularly in limitations of the activities of daily living and mobility (1, 2). Physical and occupational therapy is a key to the recovery of function (3). Effective rehabilitation requires high repetition task-oriented training, which while effective, can be labor intensive and time consuming for treating therapists (4–6). One way to address this problem is using rehabilitation robotic devices. Since the first clinical rehabilitation robot MIT-MANUS was adopted for upper limb rehabilitation, the rehabilitation robots started the era of rapid development (7). Additionally, multiple literature review papers have proved the effectiveness of robotic-assisted therapy (4, 8–10). Mehrholz's systematic review identified that the two main types of rehabilitation robot are end-effector and exoskeleton robot, the end effector provides one attachment point for the user, and the exoskeleton can align all the joints of the limb (11). There is good evidence that using rehabilitation robots in addition to usual therapy can improve recovery of motor impairments and functions in both clinical and home environments (7, 12–20). Despite this, the take up of rehabilitation robots during stroke rehabilitation is low due to the high cost, the various settings of clinical practices, accessibility for patients, and other barriers (21–23). The aim of this paper was to better understand the reasons for low levels of implementation and to inform future robot design to ensure they are feasible, safe, and acceptable for stroke survivors and professionals.

## Methods

### Study Design

**Figure 1:**
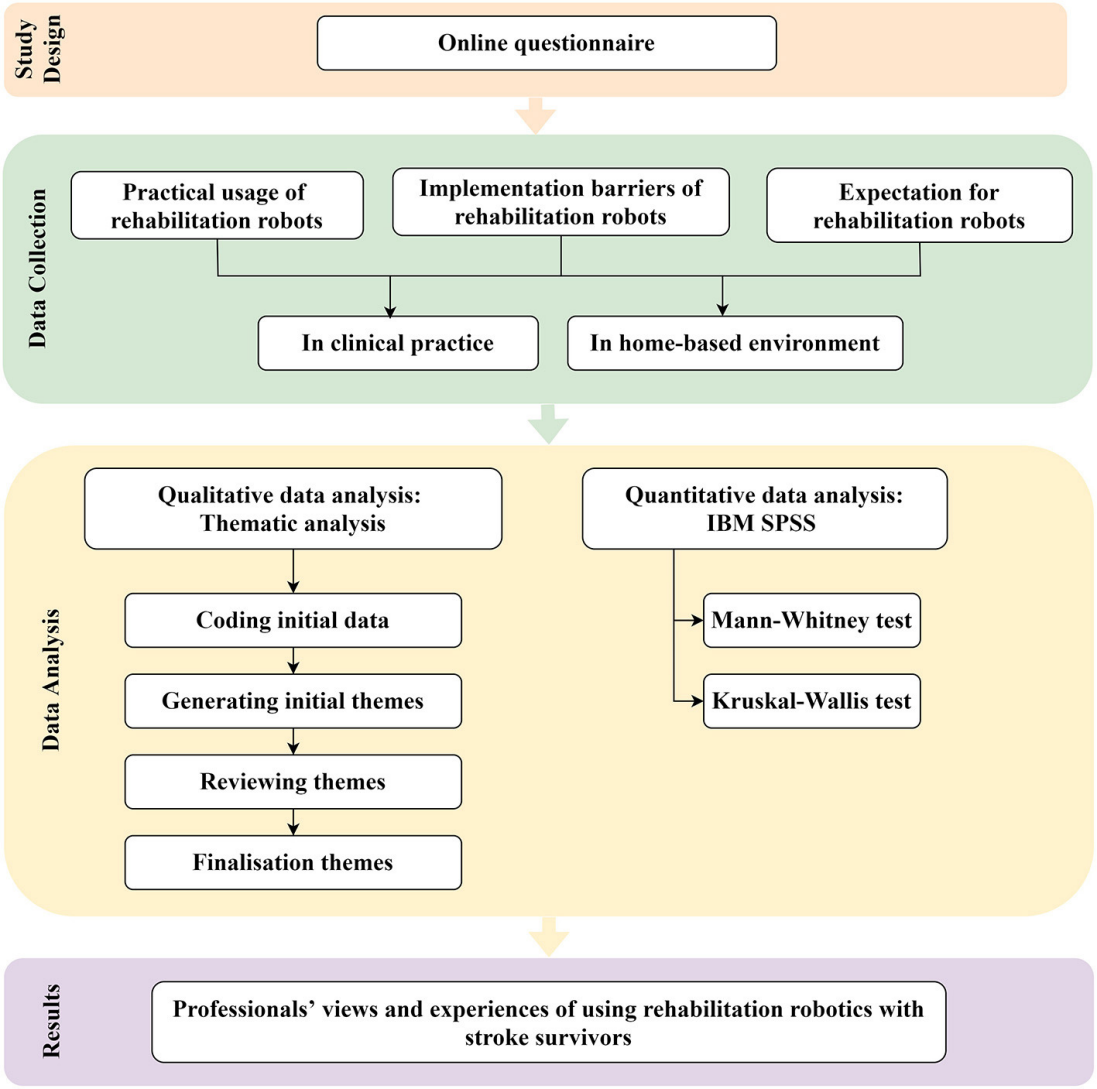
Illustration of study strategy.

In this mixed method study, an online questionnaire was devised by the authors and optimized with the support of the wider research team (see section Acknowledgment). It included three sections using a mixture of open and closed questions, the latter answered with Likert scales (Figure 1; Supplementary Material 1). The first covered the respondents' demographics, clinical experience and experience of using rehabilitation robots. The second concerned professionals' experience and views of using clinical rehabilitation robots, and the third was specifically about home-based rehabilitation robots. The questionnaire was distributed by email to the authors' clinical and relevant research networks and via social media between Novembers 2020 and February 2021 to health care professionals with current or previous experience of stroke rehabilitation (with or without use of rehabilitation robotics) in any clinical setting. A response indicated consent.

All responses were anonymous and did not contain any sensitive data thus, according to the requirement for ethical approval of the authors' institution(s), it was not required.

All quantitative data were analyzed using IBM SPSS (Statistical Package for the Social Sciences) v25.0 (IBM SPSS Statistics, RRID:SCR_019096). Descriptive statistics were used to aggregate the collected data. Mann-Whitney and Kruskal-Wallis tests compared groups. We categorized groups based on following characteristics: (1) participants' occupation (OT, occupational therapists; PT, physiotherapists); (2) career stage (<1 year, 1–5 years, 6–10 years, and >10 years); and (3) rehabilitation robot type (end-effector and exoskeleton).

For the qualitative data, an inductive thematic content analysis was undertaken using the questions in the questionnaire as a framework (16). Firstly, the respondents' statements were downloaded unedited into Excel (Microsoft Excel, RRID:SCR_016137). Then two of the authors (LT.L and ST) independently developed a coding matrix to categorize the respondents' composite statements. Our coding was shared and revised until a consensus was reached. The resulting coding framework was member checked with two respondents in the questionnaire (who had made themselves known to us—Dr. Verity Longley and Dr. Nick Preston), both experienced stroke professionals. Examples of the coding are shown in the Supplementary Material 2.

## Results

One hundred professionals responded to the survey (Table 1; Supplementary Material 3). Two-thirds were physiotherapists; one-third were occupational therapists, plus one doctor and two rehabilitation assistants. Most (71%) were from the UK, had more than 10 years of work experience (58%) and worked in the local public health system (National Health Service) (79%). Inpatient rehabilitation was the most common (78%) work environment.

**Table 1:** Respondent characteristics.

**Characteristic**	**Number (%) of participants**
**Country**	
United Kingdom	71 (71%)
Australia	6 (6%)
Europe	9 (9%)
Canada	2 (2%)
United States	2 (2%)
Brazil	1 (1%)
India	1 (1%)
New Zealand	1 (1%)
Not answered	7 (7%)
**Profession**	
Occupation therapists	35 (35%)
Physiotherapists	62 (62%)
Doctor	1 (1%)
Other (therapists assistant)	2 (2%)
Work organization^*^	
Local public health system/NHS	79 (79%)
Private practice	16 (16%)
Other (university or charity institution)	7 (7%)
Work setting^*^	
Acute care (inpatient)	63 (63%)
Inpatient rehabilitation unit	78 (78%)
Long-term care	16 (16%)
Outpatient clinical or rehabilitation facility	53 (53%)
Community-based care	62 (62%)
Other	4 (4%)
**Career stage**	
<1 year experience	6 (6%)
1–5 years' experience	20 (20%)
6–10 years' experience	16 (16%)
>10 years' experience	58 (58%)
**Highest level qualification**	
BSc/Diploma	40 (40%)
MSc/MA	30 (30%)
Extended scope/Advance care practitioner	6 (6%)
Ph.D.	15 (15%)
Other formal post-graduate qualified	8 (8%)

* *Participants indicated all the areas in which they had experience, so totals exceed 100%*.

Only 37% had experience of using rehabilitation robots, mainly in hospital and out-patient clinic environments with patients in the sub-acute stages of stroke recovery (1–3 months post stroke) (Table 2). Powered exoskeleton robots were the most commonly used type of device (Table 2). The most common dose of robotic therapy was a treatment session lasting 30–45 min, 3–5 times per week for 4–8 weeks. The most common aim of treatment for robot therapy was to increase function (89%) but increasing strength and range of movement were also frequent (84 and 76%, respectively). Respondents had used robots more frequently for upper limb rehabilitation (95%) than lower limb (25%), which tended to focus on training sagittal shoulder and elbow movements (76 and 70%, respectively) and grasp grip (51%). Approximately equal proportions of patients for whom the respondents would use a rehabilitation robot had mild, moderate or severe weakness but fewer had very severe weakness. There were no significant differences in the use of rehabilitation robotics between occupational and physiotherapists, between staff at different career stages, nor the type of robot they had used (*p* <0.05).

**Table 2:** How rehabilitation robots were used in clinical practice.

**Robotic-assisted therapy**	**Frequency (%)**
**Type of robot used**	
Exoskeleton robot with/without power supply	16 (43.2%)/10 (27.0%)
End-effector robot with/without power supply	13 (35.1%)/9 (24.3%)
Other	3 (8.1%)
**Dose of robot therapy**	
**Duration of treatment session**	
<30 min/≥30–45 min/≥45–60 min	5 (14.3%)/22 (62.9%)/8 (22.9%)
**Frequency**	
<4 weeks/≥4–8 weeks/≥8–12 weeks≥12 weeks	6 (16.7%)/19 (52.8%)/5 (13.9%)/4 (11.1%)/2 (5.6%)
**Duration of treatment**	
<3 times/week/≥3–5 times/week/≥5 times/week/No fixed frequency	8 (22.9%)/17 (48.6%)/9 (25.7%)/1 (2.9%)
**Treatment aims for robot therapy**
Increase strength	31 (83.8%)
Increase range of movement	28 (75.7%)
Increase function	33 (89.2%)
Reduce muscle tone	10 (27.0%)
Reduce/Prevent contractures	14 (37.8%)
Reduce/Prevent pain	8 (21.6%)
Other	2 (5.4%)
**Movements the robot trained**	
**Upper limb**	
Shoulder flexion or extension/abduction or adduction/internal or external rotation/horizontal abduction or adduction/multiplane movements	28 (75.7%)/18 (48.6%)/16 (43.2%)/13 (35.1%)/20 (54.1%)
Elbow flexion or extension/supination or pronation/multiplane movements	26 (43.2%)/15 (40.5%)/14 (37.8%)
Wrist flexion or extension/ulnar or radial deviation	18 (48.6%)/8 (21.6%)
Fingers flexion/or extension/abduction or adduction	16 (43.2%)/6 (16.2%)
Grasp: grasp/key or pencil/pinch/multiple	19 (51.4%)/2 (5.4%)/3 (8.1%)/4 (10.8%)
**Lower limb**	
Hip joint flexion or extension/abduction or adduction/internal or external rotation/multiplane movement	6 (16.2%)/3 (8.1%)/2 (5.4%)/3 (8.1%)
Knee flexion/extension	7 (18.9%)
Ankle dorsiflexion/plantar flexion	5 (13.5%)/3 (8.1%)
Other	1 (2.7%)
**Type of patients that have used robots—weakness**	37 (100%)
Upper limb—mild/moderate/severe/very severe	23 (62.2%)/32 (86.5%)/26 (70.3%)/8 (21.6%)
Lower limb—mild/moderate/severe/very severe	6 (16.2%)/9 (24.3%)/8 (21.6%)/5 (13.5%)
**Time after stroke that robots have been used**	37 (100%)
Acute <1 month	16 (43.2%)
Early sub-acute 1–3 months	29 (78.4%)
Late sub-acute 3–6 months	22 (59.5%)
Chronic >6 months	19 (51.4%)

Respondents with and without experience of using rehabilitation robot had similar views regarding the type of patient for whom they were suitable; patients with moderate or severe lower or upper limb weakness were considered the most suitable (Supplementary Material 4).

**Figure 2:**
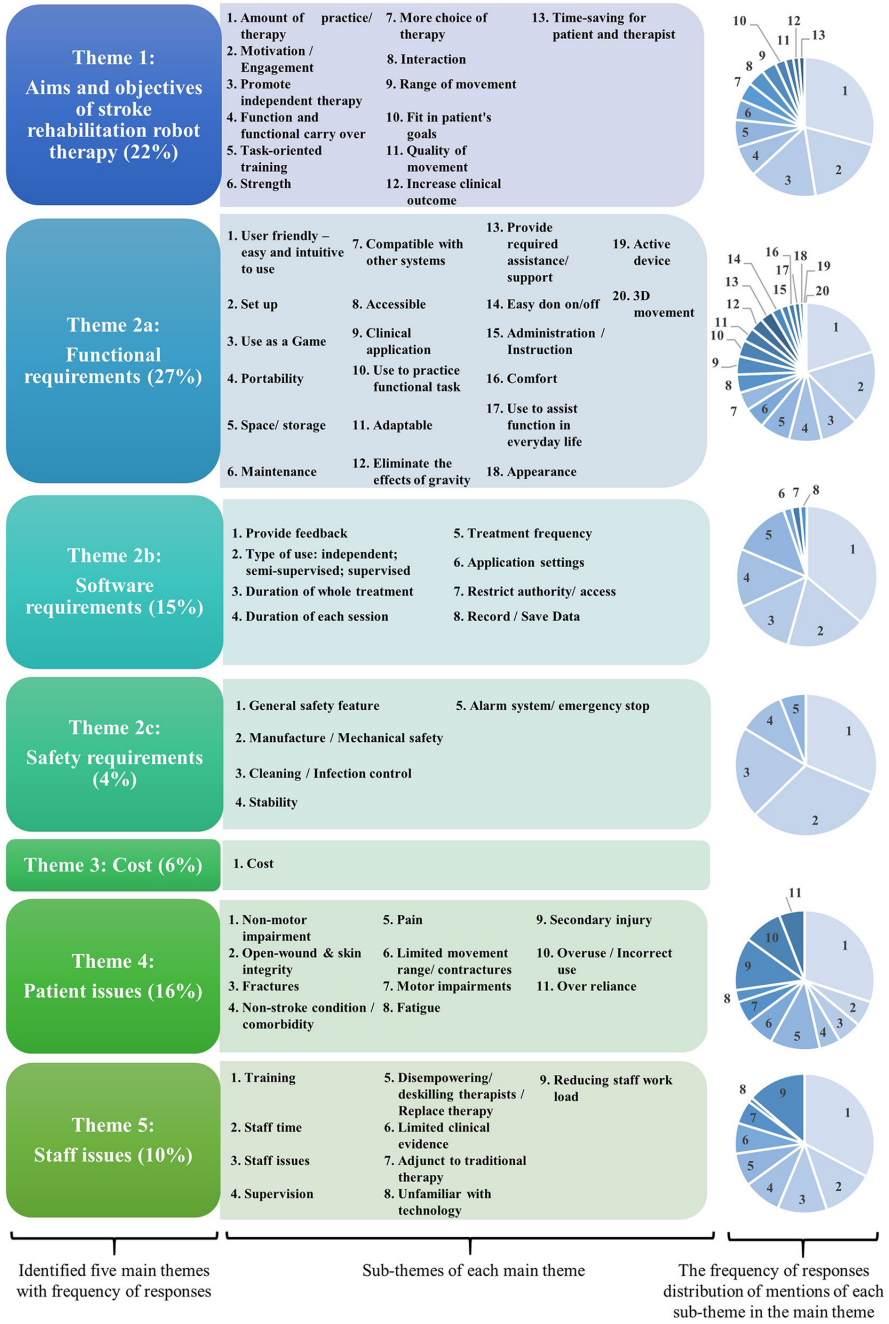
Identified themes from questionnaire data regarding rehabilitation robots in clinical and home-based settings.

Five main themes emerged from the framework analysis: “aims and objectives for rehabilitation robotics,” “requirements” (functional, software, and safety), “cost,” “patient factors” (contraindications, cautions, and concerns), and “staff issues” (concerns and benefits). These are detailed in Figure 2; Tables 3, 4.

**Table 3:** The final coding framework.

**Main theme**	**Sub-theme**	**Issue**
Aims and objectives for stroke rehabilitation robots	A rehabilitation robot needs to:	Increase clinical outcomes
		Increase the amount/intensity of practice of movements and functional activities, thereby increasing the amount of therapy provided
		Provide more choice for therapy
		Increase range of movement and strength
		Promote patients' motivation, engagement, and interaction
		Promote movement, quality of movement, task-oriented training, and functional carry-over
		Promote independent practice/patient autonomy
		Save time for patient (no travel) and therapist (semi-supervised practice)
		To assist function in everyday life
		To fit in with the patients' goals
Requirements	Functional requirements. A rehabilitation robot needs to:	Provide active and/or active assisted support to eliminate the forces of gravity in 3 Dimensions
		Be useable as a game or for functional practice
		Be highly adjustable to meet the needs of a wide range of patients' needs
		Be attractive
		Be comfortable
		User-friendly. Everything needs to be easy and intuitive to use, with as little assistance as possible: • Following instructions • Set up and programming • Donning and doffing • Adjusting/progressing program • Transport and store
		Compatible with other devices and systems e.g., clinical notes.
		Minimal maintenance in terms of time and expertise
	Software requirements. The device needs to:	Provide feedback on performance and progress to patient and/or therapist
		Record and save data: • Application settings • Usage (duration and frequency of sessions; number of repetitions) • Performance • Easy to download data to clinical notes
	Safety	Alarm system and emergency stop features needed
		Needs to fit with cleaning/infection control policies
		Stability
		Restrict access to settings, recorded data etc. so it cannot be hacked or inadvertently altered.
Cost	Cost	Minimizing cost is important. Rehabilitation robots are often assumed to be too expensive for the NHS.
Patient factors	Contraindications	Non-motor impairments e.g., behavioral problems, visual, cognitive, communication, or perceptual impairments such that the patient cannot understand or follow what needs to be done
		Non-stroke condition/comorbidity that prevents the patient being able to use the device
		Fractures
		Open-wound and skin integrity
		Patients who needs assistance which is not available (for home use)
	Cautions	Fatigue exacerbated by using the device
		Pain
		Seizures/Epilepsy if could be triggered by the screen
	Consider	Patient's willingness to use the device
		Patient and therapists' familiarity with the technology
		Risk of overuse/incorrect use/secondary injury
		Risk of patient becoming over-reliant on the technology (rely on robotic-assisted therapy to the detriment of other aspects of rehabilitation)
Staff issues	Concerns	Robotics are an adjunct to, not replacement for traditional therapy
		Robotics could disempower and deskill therapists
		The clinical evidence to support robotics is limited
		Resources—there needs to be sufficient staff and time to use the robots in addition to all other aspects of therapy
		How much supervision is needed? Can/should patients use robots independently or semi-supervised?
		Will adequate training be provided about how to operate the devices and support clinical reasoning
	Benefits	Reduces staff workload when patients can use robotics unsupervised or semi-supervised

**Table 4:** Frequency of responses to each sub-theme in different groups of respondents.

**Theme**	**Sub-theme**	**Number of mentions**										
		**Total**	**Setting**		**Experience**		**Category**					
			**Clinical**	**Home-based**	**Have used**	**Have not used**	**Advantages**	**Disadvantages**	**Requirement**	**Cautions**	**Factor**	**Usage**
Aims and objectives of stroke rehabilitation robot therapy	Amount of practice/therapy	110	82	28	49	61	97	0	4	0	8	1
	Motivation/Engagement	69	51	18	29	40	25	10	17	10	7	0
	Promote independent therapy	59	28	31	23	36	52	1	4	0	2	0
	Function and functional carry over	27	22	5	15	12	2	10	8	2	4	0
	Task-oriented training	24	22	2	6	18	16	0	8	0	0	0
	Strength	18	17	1	3	15	11	0	6	0	0	1
	More choice of therapy	16	15	1	5	11	16	0	0	0	0	0
	Interaction	15	2	13	4	11	8	7	0	0	0	0
	Range of movement	13	12	1	3	10	9	0	3	0	0	1
	Fit in patient's goals	9	9	0	2	7	0	0	8	0	1	0
	Quality of movement	7	0	7	5	2	2	1	0	4	0	0
	Increase clinical outcome	5	1	4	1	4	4	0	1	0	0	0
	Time-saving for patient and therapist	5	1	4	1	4	5	0	0	0	0	0
Requirements: Functional	User friendly—easy and intuitive to use:	95	46	49	26	58	8	21	55	7	5	3
	Set up	81	43	38	36	45	1	20	52	3	5	0
	Use as a game	42	35	6	9	32	0	0	41	0	0	0
	Portability	36	23	13	5	31	0	12	19	5	0	0
	Space/Storage	33	23	10	11	22	0	14	16	3	0	0
	Maintenance	23	11	13	10	13	0	8	8	6	1	0
	Compatible with other systems	20	10	10	5	15	0	0	20	0	0	0
	Accessible	20	11	9	11	9	9	2	7	2	0	0
	Clinical application	20	13	7	13	7	0	2	0	11	7	0
	Use to practice functional task	18	18	0	1	17	1	0	16	0	1	0
	Adaptable	15	15	0	8	7	1	0	7	0	3	4
	Eliminate the effects of gravity	14	14	0	4	10	8	0	4	1	0	1
	Provide required assistance/support	14	4	10	3	11	0	7	3	4	0	0
	Easy don on/off	11	0	11	4	7	0	0	11	0	0	0
	Administration/Instruction	8	8	0	2	6	0	5	2	1	0	0
	Comfort	7	3	4	3	4	0	0	6	0	0	1
	Use to assist function in everyday life	6	6	0	5	1	0	0	2	0	3	1
	Appearance	4	2	2	1	3	0	2	2	0	0	0
	Active device	2	1	1	0	2	0	0	2	0	0	0
	3D movement	1	1	0	1	0	0	0	1	0	0	0
Requirements: Software	Provide feedback	95	36	59	35	60	18	1	68	8	0	0
	Type of use: independent; semi-supervised; supervised	48	48	0	22	26	12	9	10	5	8	2
	Duration of whole treatment	36	36	0	36	0	0	0	0	0	0	36
	Duration of each session	35	35	0	35	0	0	0	0	0	0	35
	Treatment frequency	35	35	0	35	0	0	0	0	0	0	35
	Application settings	5	5	0	5	0	0	0	1	0	0	4
	Restrict authority/access	5	0	5	2	3	0	0	5	0	0	0
	Record/Save data	4	4	0	0	4	0	0	4	0	0	0
Requirements: Safety	General safety feature	21	4	17	3	18	0	4	16	1	0	0
	Manufacture/Mechanical safety	21	4	17	6	15	0	0	21	0	0	0
	Cleaning/Infection control	14	11	3	2	12	1	0	10	3	0	0
	Stability	7	5	2	4	3	0	1	4	2	0	0
	Alarm system/emergency stop	4	0	4	0	4	0	0	4	0	0	0
Cost	Cost	103	76	27	20	83	1	55	26	19	2	0
Patient issues: Contradictions	Non-motor impairment	86	114	2	27	59	0	5	0	78	0	2
	Open-wound and skin integrity	17	17	0	3	14	0	1	0	16	0	0
	Fractures	16	16	0	5	11	0	0	0	16	0	0
	Non-stroke condition/comorbidity	14	12	2	4	10	0	1	1	12	0	0
Patient issues: Caution	Pain	33	29	4	14	19	0	2	0	31	0	0
	Limited movement range/contractures	19	19	0	4	15	0	0	0	19	0	0
	Motor impairments	15	13	2	4	11	0	2	0	13	0	0
	Fatigue	8	5	3	3	5	0	2	0	5	0	1
Patient issues: Consider	Secondary injury	35	3	32	10	25	0	9	4	22	0	0
	Overuse/Incorrect use	26	0	26	9	17	0	10	0	16	0	0
	Over reliance	17	13	4	4	13	0	11	0	6	0	0
Staff issues: Cautions	Training	60	24	36	16	44	0	20	30	9	1	0
	Staff time	22	11	11	10	12	14	2	0	3	3	0
	Staff issues	21	17	4	13	8	3	6	6	1	4	1
	Supervision	16	8	8	4	12	1	6	2	5	2	0
	Disempowering/Deskilling therapists/replace therapy	14	6	8	3	11	0	6	0	8	0	0
	Limited clinical evidence	13	10	3	12	1	0	5	8	0	0	0
	Adjunct to traditional therapy	10	6	4	7	3	1	6	0	0	2	1
	Unfamiliar with technology	2	2	0	0	2	0	0	0	2	0	0
Staff issues: benefits	Reducing staff work load	25	24	1	7	18	25	0	0	0	0	0

Each theme is described, and the representative quotation for each theme is shown in Table 5.

**Table 5:** Representative quotation from professionals.

**Theme**	**Quotation**
Theme 1: aims and objectives of stroke rehabilitation robots	“Patients can be set up outside of therapy hours to practice independently—increases dosage” Participant 58. “I wonder if gains would translate to function without the robot assistance. I have reservations about using gaming or games. In speaking to stroke patients, rehabilitation is taken very seriously, and trivializing it is often not appreciated. I have found in practice that patients really need to clearly see the link between therapy and the impact on daily life.” Participant 45. “Promotes independence, can be done without therapist time” Participant 58. “Needs close monitoring” and “requires a qualified person with the patient” Participants 17 and 29.
Theme 2a: requirements—functional requirement	“Needs to be mobile, small as possible footprint, engaging and fun.”—participant 16.
Theme 2b: requirements—software requirements	“Patients enjoy “beating their own score”—don't think that the games need to be particularly challenging/complex” Participant 1. “Games need to be graded depending on how severe the weakness is.” Participant 13. “Games need to be age appropriate—also culturally appropriate. I think virtual reality would be good in addition to games e.g., environments you could navigate around and view things of interest”—Participant 66. “Need feedback/outcome measures which measure progress not just getting ‘better’ at the game. A larger range of games for people with significant cognitive limitations …. Games [are] often too hard to engage patients with cognitive difficulties” Participant 21. “[Need] clear reporting of results for inclusion in notes”—Participant 22.
Theme 2c: requirements—safety requirements	“Inability to accidentally change parameters set by therapist,”—Participant 22.
Theme 3: cost	“Please make cheap rehab robots available for everyone who needs one”—Participant 5. “Was effective but too expensive for the amount of effect it had.”—Participant 33. “Too expensive and NHS trusts are not funding them.”—Participant 64.
Theme 4: patient factors	“[Robot therapy] promoted progress with other deficits such as hemianopia/neglect/attentional deficits too as it elicited scanning and seceding and dividing alternating attention.”—Participant 12. “I would think extreme caution would need to be applied with patients with pain.”—Participant 47.
Theme 5: staff issues	“In practice, this would mean staffing the robot….which is not the case in reality. Therefore, most of the time, the treatment session is either/or [robot therapy or traditional therapy] rather than robot assisted training as an adjunct to 1:1 therapy time”—Participant 11. “Risk of disempowering the therapist, risk of not letting the patient develop their own motor strategies”—Participant 73. “The risk is that a therapist is not required. It empowers the patient to take charge of their rehab and have input on a daily basis. Therapists will become de-skilled and rely on robots to do their job”—Participant 78. “Needs to have evidence to back up its effectiveness”—Participant 98. “Evidence base isn't exactly overwhelmingly positive”—Participant 68.

### Theme 1: Aims and Objectives of Stroke Rehabilitation Robots

Participants reported that the main objective for using robots in stroke rehabilitation was to increase the amount of therapy patients received. This was expressed in a variety of ways; the terms “therapy,” “practice,” and “movement,” were used interchangeably as was “intensity” and “amount.” This was important, as the amount of therapy is related to the degree of recovery. Additionally, using a rehabilitation robot was thought to improve patients' motivation and engagement with therapy as it was considered an enjoyable activity. An additional objective for some participants was that using the robot enabled the patient to practice movement independently or autonomously of the therapy. However, some respondents had concerns about patients using the robots without professional supervision.

In addition, improving function and functional carry over were also important considerations, although some noted that the transfer from practicing movements with the robot to functional activity in everyday life could not be assumed. Others focused on the potential impact on impairments such as strength and range of movement. A final objective was that robotic therapy was useful to broaden the range of therapy options available to patients, which was also reported as one of the biggest advantages of using rehabilitation robots.

### Theme 2: Requirements

Respondents identified many features which were needed to ensure that a stroke rehabilitation robot was feasible and acceptable for use in clinical practice. These were categorized into functional, software, and safety requirements.

#### Functional Requirements

The participants most frequently noted that a rehabilitation robot needed to be user-friendly. It needed to be easy and intuitive to set up, administer, don and doff (put on and take off), maintain, transport, and store. The device needed to be useable as a game or to practice functional tasks/movements and be operable as either an active or an active-assisted device to eliminate the effects of gravity during three-dimensional movements. To achieve this, a robot needed to be highly adjustable to meet a wide range of patients' needs—in terms of size, level of assistance needed, cognitive demands of the games, and individual preferences. Furthermore, the appearance needed to be appealing so that it did not put patients off using it and it had to be comfortable to use.

Not only was ease of use important, but it also needed to be quick to set up, program, and “put away” as the time taken for these activities impacted on staff resources and ultimately the cost. Compatibility with other systems such as virtual reality systems or other gaming devices was also important.

#### Software Requirements

The software supporting the robot's function was considered as important as the physical device. Again this needed to be easy and intuitive to use and suitable for a wide range of needs in terms of patients' aims of treatment; level of ability, and preferences.

Participants with experience using rehabilitation robots reported that there was room for improvement with the games available. For example a greater choice and range of difficulty but also suitable for adults.

The software needed to provide feedback on usage and performance to patients and therapists to facilitate motivation and progress treatment. It was also important that the application settings, dose of use (duration, frequency, number of repetitions), and amount of assistance provided by the device were recorded and easily transferred to clinical notes. The need for data security to protect patient confidentiality was noted.

#### Safety Requirements

Safety is a paramount and non-negotiable issue for any clinical device, which was frequently raised by respondents. Their concerns and requirements went beyond the obvious need to ensure the robot did not make the patient move beyond their range of movement and was comfortable. The risk of pressure points on fragile skin or finger traps were raised, as was the need for the device to be easy to clean and to comply with infection control policies. Given the patients' movement limitations, an alarm system, emergency stop, auto shutdown, and simple/quick release mechanisms were needed to avoid incorrect or over use and to prevent secondary injury, especially for home or unsupervised use. Stability and reliability were also important issues, as was the restricting access to the settings of the robot.

### Theme 3: Cost

The high cost of rehabilitation robots and difficulty persuading hospitals to commit funds to pay for them were major barriers to their implementation into practice in both clinical and home-based settings. The cost of current rehabilitation robotics varies from $75,000 to $350,000 without any hidden cost such as taxes, installation or training, maintenance, and shipment (24). According to the “World Health Organization Choosing Interventions that are Cost-Effective” project, if a health intervention costs <3 times of the national annual gross domestic product (GDP) per capita, it will be considered as cost-effective (25). In case of UK, a robotic-assisted therapy is considered cost-effective or low-cost if the total cost is < $120,852 (update by the World Bank in December 2020) (26).

### Theme 4: Patient Factors

Participants raised many concerns and issues regarding the type of patients for whom robot therapy would not be suitable. This included people with non-motor stroke related impairments such as behavioral, cognitive, perceptual, communication, or visual difficulties which could make them unable to use or understand how to use the robot. However, one participant noted that using rehabilitation robots could be beneficial for some of these impairments, as well as motor problems, for example encouraging visual scanning and practicing memory and problem solving tasks. Further problems which could limit the patients' ability to engage with robot therapy or safety were pain, fatigue, skin integrity, spasticity, or limited range of movement, particularly fixed joint contractures and should be carefully considered before use. Co-morbidities such as fractures, other musculoskeletal problems, or epilepsy also needed to be borne in mind when considering whether robot therapy was suitable for a patient.

As well as patients' clinical condition, therapists also needed to consider the patients' and their families' willingness to try rehabilitation robots, particularly for home use. The “flip side” to this was concerns that patients could become “over-reliant” on the robots and disengage or refuse other aspects of therapy which may limit progress toward independent practice and activity in everyday life or could be a risk for overuse or secondary injuries.

### Theme 5: Staff Issues

Participants highlighted the potential impact of rehabilitation robots on staff as well as patients. A common concern was that using rehabilitation robots to deliver greater amounts of therapy could replace, disempower, or deskill therapists. Respondents were careful to point out that robotic therapy should be an adjunct, but not a substitute to “traditional” care.

Other respondents raised concerns about the resources needed to deliver robotic therapy. As therapists' working days were already full, if they were to incorporate robotic therapy into practice, either more staff were needed or they needed to stop providing some other aspect of care.

While some participants were confident to delegate the supervision of robot rehabilitation to therapy assistants (unqualified staff), family members or, for patients to use the robots unsupervised, others felt that direct supervision from qualified staff was necessary. Furthermore, several pointed out that qualified staff's time was needed to prescribe and set up the robot, problem solve, and oversee progression whether the patient was supervised or not.

Time and staffing were not the only important resourcing issue. Staff also needed adequate training and on-going support to develop clinical skills to use the robots effectively in every-day practice. As many staff regularly change work areas (referred to as “rotation”), this input needed to be on-going to accommodate staff changes. All of which increased the cost of robotic therapy.

Finally, given the professional duty to provide evidence-based care, some respondents raised the limited evidence to support its use as a barrier to implementation.

## Discussion

This research identified the benefits, requirements, concerns, and barriers to implementation of rehabilitation robots by analyzing professionals' experience and views of their use in both clinical and home-based environments. The majority (71%) of the survey responses were from UK and as such are representative of this country, it cannot be assumed that these results are necessarily representative of other geographical regions. Participants were generally positive about rehabilitation robots. Their biggest advantages was that they facilitated high repetition task-oriented training, which was consistent with the principle of efficient rehabilitation (27–30). This was achieved not only by providing physical support to enable the patient to move the weak limb but also by increasing patients' motivation and engagement to practice tasks and/or play games, which are also important factors in effective rehabilitation (13). From a professional perspective, the most commonly reported benefit for staff was the potential to reduce their work load, while providing greater amounts of therapy, as the patient could practice moving using the robot unsupervised or with “light touch” supervision, especially when the robots were home-based. However, reduced workload, and therefore cost could not be assumed, as the purchase cost of rehabilitation robots were considered prohibitive by many. Further resources included staffing and training also needed to be taken into account.

Powered upper limb robots (whether exo-skeleton or end-effector) were the most commonly used rehabilitation robot in clinical practice. This may not only be because active robots can provide a wide range of support and functions, but also because upper limb robots are relatively small, which is more convenient, than lower limb robots.

Home-based rehabilitation robots were identified as having great potential to facilitate accessibility, autonomy, and increase choice for therapy. Home-based systems can be convenient for patients as they eliminate the need to travel for access and enable autonomous usage. Professionals would be more confident in prescribing home-based robot therapy with well-developed interaction systems, such as remote monitoring and supervision.

Our findings have provided a wealth of detail regarding professionals' requirements for stroke rehabilitation robots to be fit for purpose and to be adopted into practice. In addition to reducing the costs, rehabilitation robots need to be “user friendly,” safe, and suitable for a wide range of needs. All aspects of use needed to be quick, easy, intuitive, and adaptable. Safety is, of course, a non-negotiable issue especially if the robot is to be used unsupervised. Participants recognized that although there were few absolute contraindications, robots needed to be considered cautiously for people with non-motor stroke related impairments and co-morbidities which could limit their ability to understand or to use a robot safely. Furthermore, patients' preferences also need to be taken into account—not everyone wants to play games, or use technology. However, accommodating this range of needs is likely to increase costs. Alternatively if costs are minimized, then the range of users is likely to be limited. In reality, commercialization of rehabilitation robots needs to balance these competing demands with a priority to ease of use as the mismatch between the demands of operating devices and other aspects of clinical practice has rendered many promising health technologies unfit for purpose (31–33). Future robot development work needs to consider these issues. Rather than focussing on progressing the sophistication and complexity of the technology, focussing on simpler technology and operation at lower cost may be key to successful implementation.

## Study Limitations

The main limitations of this study include the convenience of the recruitment strategies, which may be biased toward professionals with positive views or experiences of using rehabilitation robots. Although we received responses from all over the world, most were from the UK. The findings may differ if census or probability style recruitment techniques were used or if participants from other countries were involved. However, we suggest that need for low cost, user-friendly, adaptable devices is probably universal. We have also, to date, only involved professionals. Stroke survivors and their carers/families may have other views, cautions or requirements. For the further research, the involvement of these important stakeholders should be considered.

## Data Availability Statement

The original contributions presented in the study are included in the article/Supplementary Material, further inquiries can be directed to the corresponding author/s.

## Author Contributions

LL, ST, and AW conceptualized the paper. LL and ST analyzed the collected data. All authors contributed to the evaluation study itself, the manuscript, and approved the final version of the manuscript.

## Conflict of Interest

The authors declare that the research was conducted in the absence of any commercial or financial relationships that could be construed as a potential conflict of interest.

## Publisher's Note

All claims expressed in this article are solely those of the authors and do not necessarily represent those of their affiliated organizations, or those of the publisher, the editors and the reviewers. Any product that may be evaluated in this article, or claim that may be made by its manufacturer, is not guaranteed or endorsed by the publisher.
