# Supplementary material for: Professionals' Views and Experiences of Using Rehabilitation Robotics With Stroke Survivors: A Mixed Methods Survey
Source: Front Med Technol. 2021 Nov 11;3:780090. doi: 10.3389/fmedt.2021.780090 (PMC8757825; doi:10.3389/fmedt.2021.780090)
Supplement: Supplementary file 1 [file Data_Sheet_1.pdf]

Health Care Professionals' views and experience of using rehabilitation robots

Dear Therapist

Thank you for agreeing to participate in this online survey. It shouldn't take longer than ***10 to 15 minutes*** to complete. All your answers are anonymous, and will treated confidentially and stored securely.

Your response will help us understand professionals' views and experience of rehabilitation robots in different settings. This will help us develop robotic devices which are easy to use in practice, whether in clinical settings or people's homes.

The survey includes ***two sections.*** Firstly some ***background about you*** and secondly questions about ***your views and experiences of using rehabilitation robot***.

This is what we mean by ***Rehabilitation robots in clinical settings***: robotic devices which are based in hospitals or other clinical settings to which stroke patients need to travel for treatment. ***Home-based rehabilitation robots:*** the robotic device is located in the patient's home and can be used without going to the hospitals/rehabilitation centres and without direct supervision of a health care professional (although a professional may be monitoring its usage).

Part 1: Background and Treatment approach

1. What is your profession?
   ☐Occupation Therapist
   ☐Physical Therapist
   ☐Rehabilitation Nurse
   ☐Doctor
   ☐Other, please specify: ____________________

2. What is your working facility?
   ☐NHS
   ☐Private Practice
   ☐Other, please specify: ____________________

3. What is the grade/degree of you, please state?

   ____

4. In what settings have you worked with stroke clients? Please select all that apply.
   ☐Acute care (inpatient)

☐In-patient rehabilitation Unit
☐Long-term care (e.g. 3rd sector organisations; residential care)
☐Outpatient Clinic or rehabilitation facility
☐Community-based care (e.g. home-based care)
☐Other, please specify: ______________________________

5. How long have you been working with stroke clients?
☐<1 year
☐1—5 years
☐6—10 years
☐>10 years

Part 2: View and experience of using rehabilitation robots

1. Do you have any experience of using rehabilitation robot?

☐Yes, please proceed to ***Section 1***
☐No, please proceed to ***Section 2***

Section 1: For people who have USED rehabilitation robot

1.1. Could you please provide some details of the robot you have used (e.g. robot type, with or without power support, robot name if you know etc.)?

____

____

1.2. How did you used the rehabilitation robot (in terms of frequency of treatment sessions; duration of treatment sessions and overall duration of robotic treatment)?

____

____

1.3. What type of stroke patients have you used rehabilitation robot with? Please select all that apply.
☐Mild stroke ☐Moderate stroke
☐Severe stroke ☐Very severe stroke

1.4. At what stage have you used a rehabilitation robot in stroke patients? Please select all that apply.
☐<1 month after stroke ☐1—3 months after stroke
☐3—6 months after stroke ☐>6 months after stroke
☐Other, please specify ______________________________

1.5. What the treatment aim of using rehabilitation robot? Please select all that apply.
☐Upper limb rehabilitation ☐Lower limb rehabilitation

☐Other, please specify ______________________________

1.6. Which movement did the robot train?

____

____

1.7. How useful did you find the robotic device (1 is useless, 10 is most useful)?
Please choose a value: Choose an item.

1.8. How easy was the rehabilitation robot to use (1 is hardest, 10 is easiest)?
For you (the professional): Choose an item.
For the patient: Choose an item.

1.9. Were games incorporated into the robotic system?
☐Yes ☐No
If yes, how well did they work (1 is worst, 10 is best)? Please choose a value: Choose an item.

1.10. Please tell us about your thoughts / experience / recommendations about using games to support robotic training.

____

____

1.11. Did the system give feedback about patients' performance / progress?

For the professional:	☐Yes	☐No
For the patient:	☐Yes	☐No

If yes, how well did this work (1 is worst, 10 is best): Choose an item.

1.12. Did patients enjoy using the robot?
☐Yes ☐No
Please tell us about your thoughts / experiences / recommendations about this.

____

____

1.13. What do you think will be the advantages and disadvantages of using a rehabilitation robot?
Advantages: ______________________________

____

Disadvantages: ______________________________

____

1.14. Do you think rehabilitation robot increased the amount of therapy or activity the patient undertook?
☐Strongly Disagree ☐Disagree ☐Neither agree nor disagree ☐Agree ☐Strongly Agree

Please tell us about your thoughts / experiences / recommendations about this

____

____

1.15. How could the rehabilitation robot you used (or its application in practice) be improved?

1.16. Which type of patients do you think rehabilitation robot was most helpful for? Please select all that apply.

☐Mild stroke ☐Moderate stroke
☐Severe stroke ☐Very severe stroke

Please tell us about your thoughts / experiences / recommendations about this.

1.17. Which type of patients do you think rehabilitation robot was lease useful / helpful for? Please select all that apply.

☐Mild stroke ☐Moderate stroke
☐Severe stroke ☐Very severe stroke

Please tell us about your thoughts / experiences / recommendations about this.

1.18. How effective do you think the robotic device was to achieve the aims of treatment (1 is lest ineffective, 10 is most effective)?

Please select a value: Choose an item.

1.19. Is there anything else you would like us to know about you experiences or thoughts on using clinical rehabilitation robot?

Thank you please proceed to *Section 3.*

Section 2: For people who HAVE NOT Used rehabilitation robots

2.1. What types or brands of rehabilitation robots are you familiar with? If you aren't familiar with, or haven't heard of any rehabilitation robots please state 'none'.

2.2. What would be the contraindication to using a rehabilitation robot?

2.3. What aims of treatment do you think rehabilitation robot would be useful for?

2.4. How important do you think games incorporated into the robotic system (1 is least important, 10 is most important)?
Please choose a value: Choose an item.
Please tell us about your thoughts / recommendations about this

2.5. How important do you think that the robot gives you or the patient feed about the patient' performance / progress (1 is least important, 10 is most important)?
Feedback for professionals: Choose an item.
Please tell us about your thought on robotic feedback for the professional

Feedback for patients: Choose an item.
Please tell us about your thoughts on robotic feedback for the patient

2.6. What do you think would be the advantages and disadvantages of using a rehabilitation robot?
Advantages:

Disadvantages:

2.7. What concerns would you have about using a rehabilitation robot?

2.8. Which type of stroke patients do you think a rehabilitation robot may be **most** useful for? Please select all that apply.

☐Mild stroke ☐Moderate stroke
☐Severe stroke ☐Very severe stroke
Please tell us about your thoughts / recommendations about this.

2.9. At what stage do you think the rehabilitation robot may be **most** useful for patients? Please select all that apply.
☐<1 month after stroke ☐1—3 months after stroke
☐3—6 months after stroke ☐>6 months after stroke
☐Other, please specify

2.10. Which type of stroke patients do you think a rehabilitation robot may be **least** useful for? Please select all that apply.

☐Mild stroke ☐Moderate stroke

☐Severe stroke ☐Very severe stroke

Please tell us about your thoughts / recommendations about this.

2.11. At what stage do you think the rehabilitation robot may be **least** useful for patients? Please select all that apply.

☐<1 month after stroke ☐1—3 months after stroke

☐3—6 months after stroke ☐>6 months after stroke

☐Other, please specify

2.12. Is there anything else you would like us to know about you thoughts or recommendations on using clinical rehabilitation robot?

Section 3. We are interested in the possibilities of producing a rehabilitation robot that patients could use in their own homes to increase the amount of therapy/practice/activity they can undertake.

We envisage that after setting up the device and teaching the patient how to use it, the treating health care professional would provide 'light touch' indirect monitoring, advice and progression but the patient would use the device without direct supervision. ***All following questions are based on this condition.***

3.1. Would you be interested in using a rehabilitation device in this way (1 is not interested, 10 is very interested)?

Please choose a value: Choose an item.

3.2. What would be the advantages and disadvantage of using a rehabilitation robot in this way?

Advantages:

Disadvantages:

3.3. What concerns would you have about patients using a rehabilitation robot in this way?

3.4. Which types of patients would it be suitable for? Please select all that apply.

☐Mild stroke ☐Moderate stroke

☐Severe stroke ☐Very severe stroke

Please tell us about your thoughts / recommendations about this.

3.5. Which types of patients would it **NOT** be suitable for? Please select all that apply.

☐Mild stroke ☐Moderate stroke
☐Severe stroke ☐Very severe stroke

Please tell us about your thoughts / recommendations about this.

____

____

3.6. What aims of treatment would be suitable for using robot in this way?

____

____

3.7. What features of the rehabilitation robot would be necessary or helpful to make you more confident to use a rehabilitation robot without direct supervision (e.g. safety features? appearance? Feedback options)

____

____

3.8. What features of the rehabilitation robot would be necessary or helpful to make it easier for the patient to use?

____

____

3.9. Is there anything else you would like us to know about your experience or thoughts on using home-based rehabilitation?

____

____

ALL DONE! Thanks again for your participation!

If you have anything would like to discuss with us, please contact with Lutong Li:
lutong.li@manchester.ac.uk
