# Supplementary material for: Professionals' Views and Experiences of Using Rehabilitation Robotics With Stroke Survivors: A Mixed Methods Survey
Source: Front Med Technol. 2021 Nov 11;3:780090. doi: 10.3389/fmedt.2021.780090 (PMC8757825; doi:10.3389/fmedt.2021.780090)
Supplement: Supplementary file 2 [file Data_Sheet_2.docx]

**Supplementary material 2. Examples of how respondents’ statements were coded**

| **Questionnaire quotation** | **Initial coding framework** |
| --- | --- |
| “Patient understanding, challenging behaviour (precaution), pain, available range (if this falls outside the adjustable parameters of that robot), size (i.e. not fitting into the device), seizure risk if it involves a screen in patients with photosensitive epilepsy, open wounds that cannot be covered” | Patient factors   - Behaviour - Cognition - Pain - Limited range of movement - Open wounds   Requirements   - Functional - Adjustable - Safety |
| “With the right patient it can significantly increase repetitions through engagement with a game or enabling the use of a movement otherwise inaccessible to that patient. However, they are often very time consuming to set up, some patients do not want to use them and they are usually VERY expensive!” | Aims   - Increase repetitions/ amount of therapy/practice - Increase engagement - Increase amount of movement   Functional requirements   - Set up: time-consuming   Cost |
| “In some patients it was successfully used as an adjunct and increased practice. In other cases, it replaced or was used alongside other upper limb rehab (e.g., of 5 upper limb session, 2 were using Armeo, or it was the most successful intervention at engaging the patient). Time and staff resources were main limiting factors.” | Aims   - Increase the amount of practice - Engagement   Staff Issues   - Adjunct to therapy - Need enough staff and time |
| “Usually ends up taking from something else, so overall provision is the same but perhaps intensity when using the robot is higher than normal care. Sometimes difficult for patients to see how the robot activities transfer to normal day to day activities to improve carryover and longer lasting change” | Aim   - Greater intensity of practice /therapy   Staff issue   - An adjunct not replacement - Time – staff don’t have spare time so it detracts from other aspects of therapy   Functional requirements   - Needs to be functional to transfer to everyday |
