# Supplementary material for: Professionals' Views and Experiences of Using Rehabilitation Robotics With Stroke Survivors: A Mixed Methods Survey
Source: Front Med Technol. 2021 Nov 11;3:780090. doi: 10.3389/fmedt.2021.780090 (PMC8757825; doi:10.3389/fmedt.2021.780090)

Supplementary material 4. Respondents' responses to the question "Which type of patients do you think rehabilitation robots will be most helpful for?"

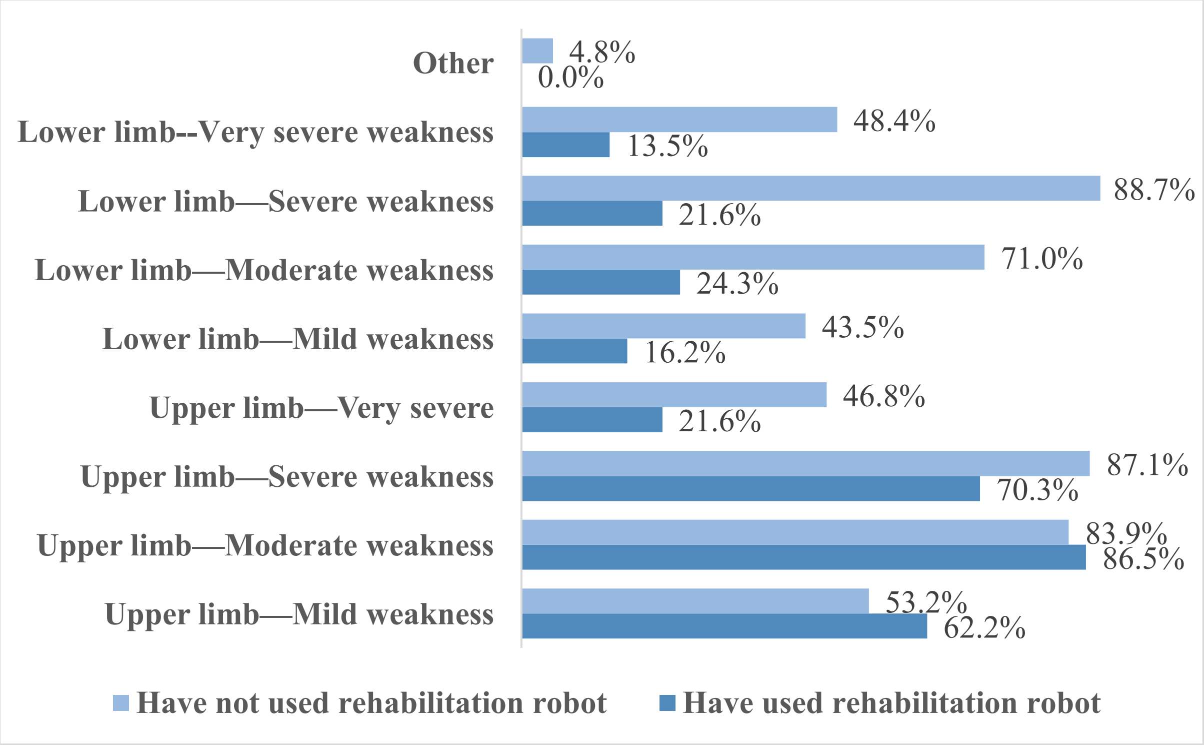

Supplement: Supplementary file 4 [file Data_Sheet_4.pdf]
